# Knockout of Auxin Response Factor SlARF4 Improves Tomato Resistance to Water Deficit

**DOI:** 10.3390/ijms22073347

**Published:** 2021-03-25

**Authors:** Mengyi Chen, Xiaoyang Zhu, Xiaojuan Liu, Caiyu Wu, Canye Yu, Guojian Hu, Lin Chen, Riyuan Chen, Mondher Bouzayen, Mohammed Zouine, Yanwei Hao

**Affiliations:** 1 Key Laboratory of Horticultural Crop Biology and Germplasm Innovation in South China, Ministry of Agriculture, College of Horticulture, South China Agricultural University, Guangzhou 510642, China; mengyi_chen@126.com (M.C.); xiaoyang_zhu@scau.edu.cn (X.Z.); 17379756187@163.com (X.L.); wucaiyu@stu.scau.edu.cn (C.W.); cyyu@stu.scau.edu.cn (C.Y.); hu.guojian0309@gmail.com (G.H.); chen07041@126.com (L.C.); rychen@scau.edu.cn (R.C.); 2 Institute of Bioengineering, Guangdong Academy of Sciences, Guangzhou 510316, China; 3 Laboratory Genomics and Biotechnology of Fruits, INRA, Toulouse INP, University of Toulouse, 31320 Castanet Tolosan, France; mondher.bouzayen@toulouse-inp.fr

**Keywords:** SlARF4, tomato, water deficit, drought, ABA, auxin

## Abstract

Auxin response factors (ARFs) play important roles in various plant physiological processes; however, knowledge of the exact role of ARFs in plant responses to water deficit is limited. In this study, SlARF4, a member of the ARF family, was functionally characterized under water deficit. Real-time fluorescence quantitative polymerase chain reaction (PCR) and *β*-glucuronidase (GUS) staining showed that water deficit and abscisic acid (ABA) treatment reduced the expression of *SlARF4*. *SlARF4* was expressed in the vascular bundles and guard cells of tomato stomata. Loss of function of SlARF4 (*arf4*) by using Clustered Regularly Interspaced Short Palindromic Repeats/Cas 9 (CRISPR/Cas 9) technology enhanced plant resistance to water stress and rehydration ability. The *arf4* mutant plants exhibited curly leaves and a thick stem. Malondialdehyde content was significantly lower in *arf4* mutants than in wildtype plants under water stress; furthermore, *arf4* mutants showed higher content of antioxidant substances, superoxide dismutase, actual photochemical efficiency of photosystem II (PSII), and catalase activities. Stomatal and vascular bundle morphology was changed in *arf4* mutants. We identified 628 differentially expressed genes specifically expressed under water deficit in *arf4* mutants; six of these genes, including ABA signaling pathway-related genes, were differentially expressed between the wildtype and *arf4* mutants under water deficit and unlimited water supply. Auxin responsive element (AuxRE) elements were found in these genes’ promoters indicating that SlARF4 participates in ABA signaling pathways by regulating the expression of *SlABI5/ABF* and *SCL3*, thereby influencing stomatal morphology and vascular bundle development and ultimately improving plant resistance to water deficit.

## 1. Introduction

Originally a native of Central America, tomato (*Solanum lycopersicum* L.) has become one of the most important economic crops in the world. Tomato growth and development requires ample water supply, and water stress can indeed severely limit germination, radicle and hypocotyl elongation, and overall biomass accumulation [1,2,3,4]. However, tomato plants respond to a shortage in water supply by adjusting their morphology and physiological and biochemical processes. Physiological response mechanisms to water deficit include modifying diverse traits, such as increasing the thickness of the epidermal epicuticular wax layer, adjusting stomata closure, favoring root elongation over aboveground biomass allocation, strengthening the water storage machinery and reducing shoot to root ratio, among other things [2,3,5,6]. Concomitantly, at the biochemical level, tomato plants experiencing water deficit produce osmotic regulators, notably increase the activity of protective enzymes and procure the stability of membrane systems; additionally, they unleash the regulatory action of endogenous hormones and the synthesis of secondary metabolites that participate in the defense to prevent or minimize potential damage [7,8,9,10]. These physiological and biochemical changes are caused by stress-induced plant hormones such as abscisic acid (ABA), various proteins such as the late embryo-enrichment protein and the tomato dehydration protein [8,11,12,13], and the NAC (NAM, ATAF1/2, and CUC2) transcription factor [9] as well as various small RNAs [14] that regulate tomato water stress resistance-related gene expression aiming to reduce the damage by water deficit to plant growth and development.

The auxin response factor (ARF) has been suggested to play a key role in regulating the expression of auxin responsive genes [15,16,17]. ARFs combine with Auxin responsive element (AuxRE) elements located in the promoter region of auxin responsive genes to regulate their transcription and further regulate plant growth and metabolism [15]. Genes of the ARF family have been found in herbaceous plants such as Arabidopsis, rice, corn, and tomato, and among woody plants such as poplar, eucalyptus, and tea [18,19,20,21,22]. ARFs have been implicated in senescence [23], hormone signaling [11,24,25] and developmental programs [11,23,26]. In rice, OsGSK5/OsSK41 interact with OsARF4 to negatively regulate grain size and weight [27]. In Arabidopsis, *ARF2*, together with the Homeodomain Gene *HB33* mediate ABA responses [24]; in turn, MP/ARF5 function upstream of the AtHB8 to regulate embryo development, as well as vascular differentiation [28]. However, to date, the role of ARFs in plant responses to water stress is scarcely understood. The expression levels of multiple ARF genes were altered in tomato by drought stress [29]. There are 24 ARF genes in tomato [22], which have an effect on plant growth and development, including fruit set [30,31,32,33], root development [34], leaf morphology [35], and fruit ripening [36,37]. *SlARF5*, *SlARF7*, and *SlARF8* regulate fruit set and parthenocarpy by mediating auxin- and gibberellin GA-signaling pathways [30,31,32,33]. *SlARF2* controls fruit ripening by mediating ethylene and ripening transcription regulators [37]; additionally, *SlARF2* regulates tomato root development [34]. Finally, *SlARF4* and *SlARF10* increase chlorophyll and sugar accumulation during fruit development [38,39]. However, there are very few reports on the functional description of ARF in relation to resistance to water deficit.

Therefore, in this study, physiological, biochemical and molecular biology methods were used to study the function of SlARF4 in mediating tomato resistance to water deficit aiming to understand the role of ARFs in tomato drought resistance. The results showed that knockouts of SlARF4 using Clustered Regularly Interspaced Short Palindromic Repeats/Cas 9 (CRISPR/Cas 9) resulted in induced water deficit resistance and rehydration ability. The ABA signaling pathway gene *SlABi5/ABF* were upregulated in the *arf4* mutant. Transcription factor SCL3, also named GRAS4, induced tomato drought resistance by modulating the ABA signaling pathway. In our data, we found SlARF4 could directly regulate *SCL3* expression. Altogether, our data indicate that *SlARF4* participates in ABA signaling pathways by regulating *SlABI5/ABF* and *SCL3* expression and operating the morphology of stomata and development of vascular bundles to improve plant resistance to water deficit.

## 2. Results

### 2.1. SlARF4 Expression Is Downregulated in Response to Abscisic Acid (ABA) and Water Deficit

**Figure 1:**
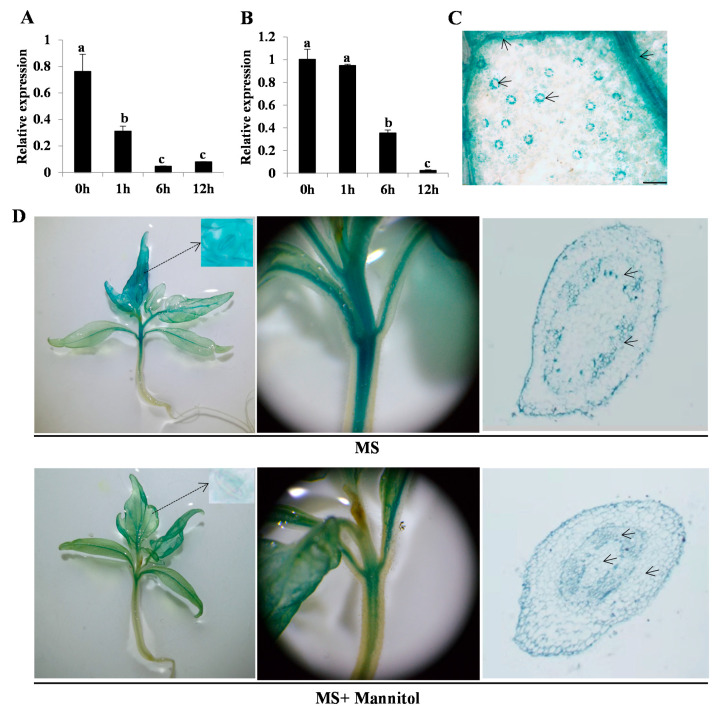
Expression pattern of *SlARF4*. Results of quantitative real-time polymerase chain reaction (qRT-PCR) analysis showing that *SlARF4* expression is reduced by (**A**) water deficit and (**B**) abscisic acid (ABA) treatment. Four-week-old tomato seedlings were treated with 100 μM ABA or by desiccation for different durations; whole seedlings were used for RNA extraction. Polyubiquitin (UBQ) (Solyc01g056940) was used as an internal standard. Data was means ± standard error (SE) of three independent biological replicates. Different letters (a, b, c) presented significant difference at level set *p* < 0.05; (**C**) Detection of *SlARF4* promoter activity in tomato leaves by histochemical GUS staining. (**D**) Detection of *SlARF4* promoter activity in tomato seedlings under normal and water stress conditions. Two-week-old seedlings harboring the pARF4::GUS transgene grown in MS and MS plus mannitol media were subjected to GUS staining. Aboveground part of aseptically cultured pARF4::GUS seedlings grown for 3 weeks (**left**); enlarged view of the middle and upper part of the stem (**center**), paraffin section of the stem observed microscopically at 10 (**right**). Black arrow indicates the stomata and xylem. MS medium; MS + 100 μM mannitol medium. The dot arrows indicates the stomata.

We measured *SlARF4* expression levels in tomato seedlings under ABA and water deficit treatments to determine whether SlARF4 expression is reduced by either. Results of quantitative real-time polymerase chain reaction (qRT-PCR) analysis revealed that the expression level of *SlARF4* showed a decreasing trend with increasing duration of water deficit (Figure 1A). The expression level was the lowest at 6 h of treatment initiation and slightly increased at 12 h (Figure 1A). Furthermore, as duration of ABA treatment increased, the expression of *SlARF4* decreased (Figure 1B).

Results of *β*-glucuronidase (GUS) staining showed that *SlARF4* was expressed in the guard cells of the stomata and in leaf vascular tissues (Figure 1C). In GUS staining, 2-week-old seedlings subjected to water stress were dyed. The accumulation of GUS in pSlARF4::GUS seedlings grown in Murashige & Skoog (MS) medium containing mannitol was significantly lower than that observed in MS medium alone (Figure 1D). Paraffin sections of hypocotyls of both seedlings showed that GUS mainly accumulated in the xylem, epidermis and guard cells (Figure 1D).

### 2.2. Knockout of SlARF4 by CRISPR/Cas 9 Increased Plant Sensitivity to Water Stress

**Figure 2:**
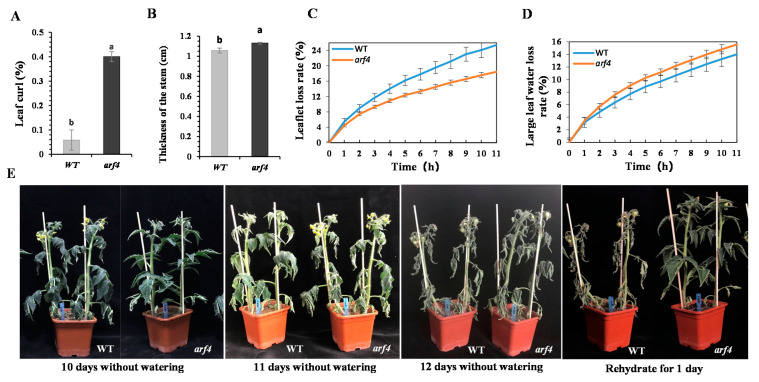
Effects of *SlARF4* knockout on resistance to water deficit, morphology, and transpirational water loss in tomato plants. (**A**) Degree of curliness of 2-month-old tomato leaves; (**B**) Thick stem of two-month-old tomato plants. (**C**) Rate of water loss from small leaves isolated from 2-month-old tomato plants at room temperature. (**D**) Rate of water loss from mature leaves excised from 2-month-old tomato plants at room temperature. (**E**) Effect of water deficit on wild-type (WT, left) and *arf4* mutant (right) tomato plants. Data was means ± SE of three independent biological replicates. Different letters (a, b) presented significant difference at level set *p* < 0.05.

SlARF4 knockout mutants showed conspicuous upward leaf-curling (Figure 2A) and a stem thickness (Figure 2B) significantly greater than that of the wild-type (WT) plants. Additionally, the rate of water loss from *arf4* leaflets was lower than that of the corresponding leaves in the WT plants (Figure 2C), whereas the rate of water loss in *arf4* mature leaves was higher than that of the corresponding leaves in the WT plants (Figure 2D). Both WT and *arf4* plants appeared wilted at 12 days after water-stress treatment initiation (Figure 2E). However, leaves of *arf4* plants were upright again after 24 h of re-watering, whereas those of the WT plants were still wilted (Figure 2E). From a morphological point of view, *arf4* plants had basically recovered. Different concentrations of mannitol dissolved in MS medium simulated water stress to verify the effect of water deficit on the germination rate of tomato seeds. The results showed that the higher the mannitol concentration, the lower the germination rate of WT seeds, whereas *arf4* seeds still maintained a high germination rate even at 300 μM mannitol, which was 58% higher than that of WT seeds (Figure S1A). Concomitantly, *arf4* plants showed longer hypocotyls and roots (Figure S1B–D).

**Figure 3:**
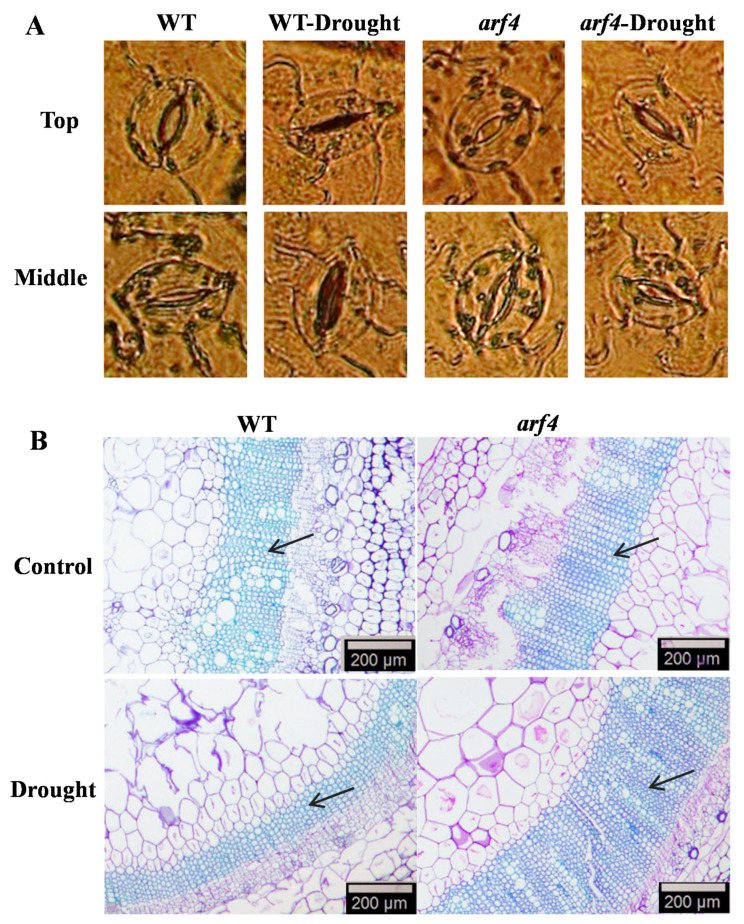
Morphology of stomata on tomato leaf epidermis (**A**) and paraffin section of a 2-month-old tomato stem (**B**). The black arrow represents the xylem. The scale represents 200 μm.

Under unlimited water supply, as well as under conditions of water stress, stomata and guard cell length on the upper leaves of *arf4* plants was significantly shorter than that on the same leaves in the WT plants and tended to be more rounded (Table S1). Water stress induced stomatal closure in the WT plants, whereas the stomates of *arf4* plants did not close normally (Figure 3A). Similarly, the stomates of epidermal cells in the middle leaves of *arf4* plants were wider and rounder, whereas those of the WT plants appeared shriveled under water stress (Figure 3A). SlARF4 absence changed stomatal morphology and reduced the length of the stomates, and the stomata did not close normally under water stress. The stem xylem of 2-month-old tomato WT and *arf4* plants showed a dense and compact arrangement under water stress, with the xylem in *arf4* being more compact and the xylem range wider (Figure 3B).

### 2.3. Leaf Damage and Physiological Changes of *arf4* Plants under Water Deficit

**Figure 4:**
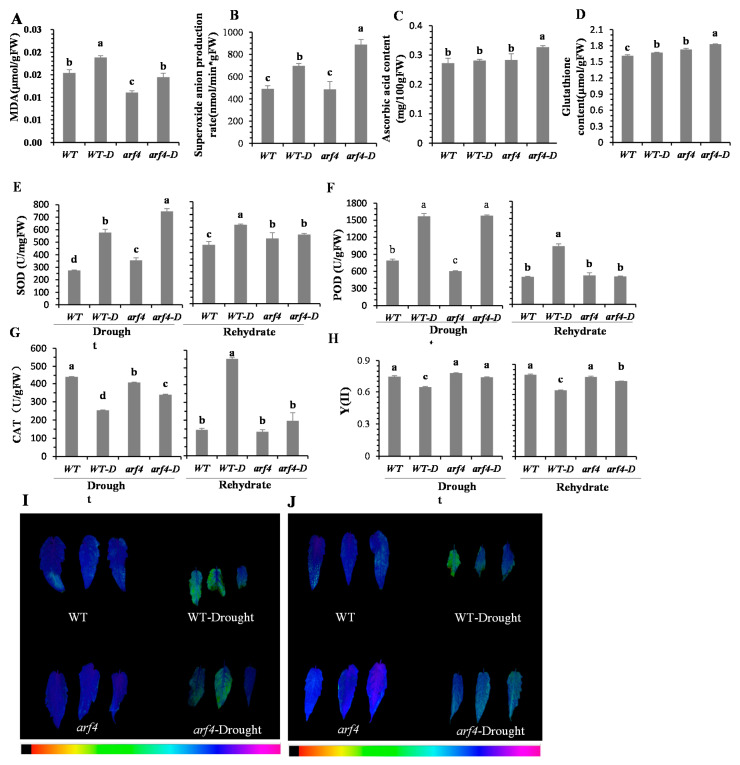
Effect of water stress on antioxidant capacity of WT and *arf4* mutant tomato leaves. (**A**) malondialdehyde (MDA) content; (**B**) superoxide anion production rate; (**C**) ascorbic acid content; (**D**) glutathione content; (**E**) superoxide dismutase (SOD) activity of plant under 12 days water deficit and 1 day of re-watering; (**F**) peroxidase (POD) activity of plant under 12 days water deficit and 1 day of re-watering; (**G**) catalase (CAT) activity of plant under 12 days water deficit and 1 day of re-watering; (**H**) the actual photochemical efficiency of PSII (YII) of plant under 12 days water deficit and 1 day re-watering. (**I**) The maximum photochemical efficiency of PSII (Fv/Fm) was determined on well watering plant and plant with 12 days water deficit. The underneath color code depicted in the image ranges from 0 (black) to 1 (purple); (**J**) The maximum photochemical efficiency of PSII (Fv/Fm) was determined on well watering plant and plant after one day of re-watering. The underneath color code depicted in the image ranges from 0 (black) to 1 (purple). Different letters (a, b, c, d) present significant difference at level set *p* < 0.05. WT-D and arf4-D represent wild-type (WT) and arf4 under water deficit, respectively.

Water stress reduces plant water content; additionally, it triggers the accumulation of reactive oxygen species (ROS) and malondialdehyde (MDA); furthermore, it causes biofilms to oxidize and reduces photosynthetic capacity. Superoxide anion production rate in *arf4* plants under water stress was significantly higher than that in WT plants (Figure 4B), whereas MDA content a proxy of cell membrane damage was significantly lower in *arf4* plants than in WT plants (Figure 4A). *arf4* plants showed a high level of antioxidant enzyme activities, including of superoxide dismutase (SOD), peroxidase (POD) and catalase (CAT) (Figure 4E–G), and antioxidants such as ascorbic acid and glutathione (Figure 4C,D), which effectively reduced oxidative damage in leaves. The actual photosynthetic efficiency of *arf4* plants did not decrease significantly due to water stress (Figure 4H). Consistently, maximum fluorescence intensity of tomato leaves showed that WT leaves exhibited partially irreversible damage under water stress, whereas *arf4* leaves remained intact (Figure 4I). To understand the state of *arf4* plants at the physiological level after re-watering, SOD, POD, and CAT activities were restored to the normal level (Figure 4E–G), while actual photosynthetic efficiency was significantly higher than that in the WT plants (Figure 4H), and fluorescence intensity filled the entire leaf, but the WT plants could not return to normal after 1 day of re-watering (Figure 4J).

### 2.4. Experimental Design for Transcriptomic Analysis of Wild-Type (WT) and arf4 Leaves under Water Stress and Unlimited Water Supply

Compared with WT plants, *arf4* mutants were more resistant to water stress. RNA-seq was carried out on WT and *arf4* mutant leaves under unlimited water supply and under water stress conditions to further understand the genes or gene networks involved in the regulation of resistance to water deficit. The complete experimental design included four parallel experiments. The first experiment was conducted to identify the genes whose expression was associated with water deficit in *arf4* plants. The second experiment was performed to identify the genes related to water stress response in the WT plants. The third experiment was conducted to identify the genes directly or indirectly regulated by *SlARF4* in WT and *arf4* leaves under unlimited water supply. Finally, the fourth experiment was performed to identify the genes directly or indirectly regulated by *SlARF4* in WT and *arf4* leaves of plants kept under conditions of water deficit. For this purpose, we conducted a comprehensive analysis of gene expression in WT and *arf4* leaves under unlimited water supply and under water deficit: WT, *arf4*, WT-D, and *arf4*-D. All samples contained three biological replicates and generated 12 libraries. The high-quality clean reads of the library reached over 98% (Table S2). After filtering the rRNA, the library was uniquely mapped to the tomato genome (*Solanum lycopersicum* ITAG2.3). The mapped reads ranged between 90.55% and 93.15% (Table S2). Unique mapped reads ranged from 89.96% to 92.38% and multiple mapped reads on the reference genome accounted for 0.54% to 0.80% (Table S2). According to the fragments per kilobase per million (FPKM) method and the Pearson correlation coefficient (R^2^ > 0.8 indicates a significant correlation between the two samples), R^2^ among the three replicates was greater than 0.94 (Figure S2), and the biological replicates were significantly correlated (Figure S2). Therefore, sequencing results were highly reliable.

**Figure 5:**
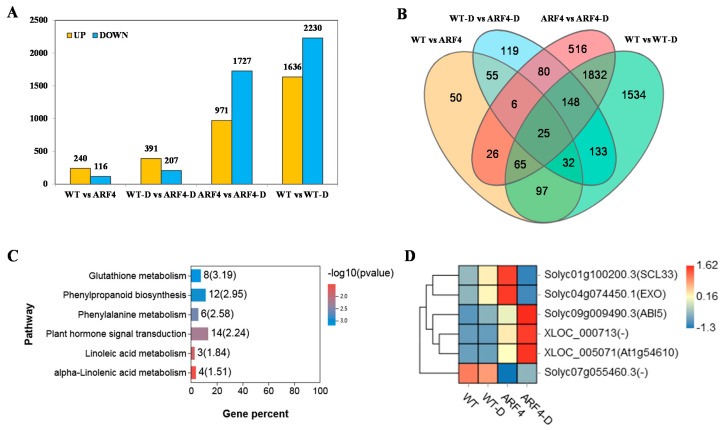
Analysis of differential gene expression between WT and arf4 mutant tomato plants. (**A**) Numbers of differentially expressed genes (DEGs) between WT and *arf4* mutants under unlimited water supply and under water stress conditions, (**B**) overlap of these genes set. (**C**) Kyoto Encyclopedia of Genes and Genomes (KEGG) analysis of DEGs specifically expressed in *arf4* plants under water stress and (**D**) heatmaps of six DEGs differently expressed between WT and *arf4* plants under conditions of unlimited water supply and water stress. WT and *arf4* under water stress are represented by WT-D and arf4-D (auxin response factor ARF4-D), respectively.

We performed a comprehensive analysis of gene expression related to the response to water stress in WT and *arf4* leaves, aiming to identify candidate genes that are vital for induction of resistance to water deficit. Genes that satisfied the fold-change difference |log2 (fold-change)| >1 and false discovery rate (FDR) <0.05 were regarded as differentially expressed genes (DEGs). Deseq2 software was used for performing pairwise comparisons to screen DEGs. In all, 2689 (971 upregulated + 1727 downregulated) and 3866 (1636 upregulated + 2230 downregulated) DEGs between *arf4* mutants and WT plants, respectively, were involved in the response to water stress (Figure 5A). A total of 628 (516 + 80 + 6 + 26) DEGs were specifically expressed in *arf4* mutants under conditions of water deficit (Figure 5B); among them, 86 showed SlARF4-dependent regulation in WT and *arf4* leaves under water deficit (Figure 5B), 33 more showed SlARF4-dependent regulation in WT and *arf4* leaves under conditions of unlimited water supply (Figure 5B), and 6 more DEGs showed SlARF4-dependent regulation in WT and *arf4* leaves under conditions of both water stress and unlimited water supply (Figure 5B).

### 2.5. Transcriptome Analysis of Response of Differentially Expressed Genes (DEGs) to Water Deficit in Tomato WT and *arf4* Mutant Plants

According to Venn diagram analysis (Figure 5B), 628 DEGs were found specifically expressed in *arf4* mutants under water stress. We hypothesized that the expression of these genes may play an important role in improving the resistance of *arf4* mutants to water deficit. To gain further insight into the putative functions of these genes, all 628 DEGs were mapped to the Kyoto Encyclopedia of Genes and Genomes (KEGG) database, which revealed that 105 of these DEGs were assigned to 69 KEGG pathways (Supplementary Table S3). Using *q* value < 0.05 as significance threshold, six KEGG pathways were significantly enriched, namely, “glutathione metabolism,” “plant hormone signal transduction,” “phenylpropanoid synthesis,” “phenylalanine metabolism,” “alpha-linoleic acid metabolism,” and “linoleic acid metabolism” (Figure 5C). Hormone signaling pathways mainly involved auxin-, cytokine-, abscisic acid (ABA)-, jasmonic acid-, and salicylic acid (SA) signal transduction pathways (Figure S3). Upregulation of multiple genes in phenylpropanoid biosynthesis is shown in Figure S4. The phenylpropanoid biosynthesis pathway is mainly related to the synthesis of lignin, which affects the development of vascular bundles.

Among the 628 DEGs specifically expressed in *arf4* mutants, 33 genes were differentially expressed in WT and *arf4* mutant plants under normal conditions, and all 33 were mainly involved in 12 pathways (Supplementary Table S5). Among them, the change in major hormone signaling pathway-related gene expression involved the ABA signaling pathway (Figure S5). Among the 628 specifically expressed genes of *arf4* mutants, 86 genes were differentially expressed between WT and *arf4* mutant plants under water deficit, and these 86 genes were mainly involved in 17 pathways (Supplementary Table S4). Among them, phenylpropanoid biosynthesis, hormone signaling pathway, and α-linolenic acid metabolism (JA synthesis) included 2 to 3 DEGs. In the hormone signaling pathway, the expression of two genes of ABA and SA signaling pathways changed (Figure S5), indicating that the ABA and SA signaling pathways are involved in the response of *arf4* mutants to water deficit, in which case, ABA signaling may play an important role.

### 2.6. Analysis of Six DEG Promoters between WT and *arf4* Plants and Their Relationship with the ARF4 Protein

Among the 628 genes specifically expressed in *arf4* mutants, six DEGs were differentially expressed between WT and *arf4* mutant plants under conditions of unlimited water supply and under water deficit stress (Figure 5D). Among them, there were two unknown genes (XLOC_000713 and XLOC_005071) that were not annotated to the tomato genome, whereas the remaining four genes were *ABI5*, *SCL3* (*GRAS4)*, *EXO* and *Phi-1*(*solyc07g055460.3)*. The 3000-bp upstream promoter region of these four genes was obtained from the tomato genome database (https://solgenomics.net) (accessed on 26 January 2021). Cis-element analysis of these promoter regions revealed that *ABI5*, *EXO*, and *Phi-1*(*solyc07g055460.3*) have one AuxRE cis-acting elements; and *SCL3(GRAS4)* has two AuxRE cis-acting elements, indicating that the expression of these four genes can be directly regulated by ARF4 (Figure S5). Two genes, *ABI5* and *SCL3(GRAS4)* were involved in ABA signal transduction pathways indicating that SlARF4 regulates tomato resistance to water stress by regulating ABA signaling pathways.

### 2.7. Validation of RNA-seq Data by Quantitative Real-Time Polymerase Chain Reaction (qRT-PCR)

To investigate the accuracy and reproducibility of the RNA-seq data, 13 DEGs were selected from RNA-seq results for qRT-PCR (Figure S6). Using *SlUBI* as internal reference gene, the 2^−ΔΔCT^ method was used to calculate relative gene expression. qRT-PCR findings for the 13 selected genes were consistent with the RNA-seq results, revealing high accuracy and reliability of our RNA-seq results. Transcriptome results were normalized to FPKM by the Z-score, and the Pearson correlation coefficients for the two were R^2^ > 0.8 and *p* < 0.01 (Figure S7), indicating that our transcriptome sequencing results are reliable.

## 3. Discussion

Leaf curling is an important plant response to characterize resistance to water deficit. Auxin polar transport plays an important role in the establishment of leaf proximal-distal axis polarity [40]. Changes in the asymmetry of the axial development of leaves in higher plants will cause leaves to curl [41,42]. Here, we showed that the lack of SlARF4 resulted in obvious leaf curling phenotypes in tomato plants. Leaf curling can reduce effective leaf area and, consequently, transpirational water loss, thereby increasing the ability of the plant to improve water-use efficiency [43,44]. In natural environments, stress factors do not occur independently but in combination; thus, water deficit is frequently concomitant with high temperature. Heat stress can cause tomato leaves to curl and wilt [44,45]. However, reports on leaf curling caused by water deficit are relatively rare. In rice, wheat, corn, and other plant species, leaf curling is considered to enhance resistance to water deficit [44]. As the stress duration increases, leaf curling in *Ctenanthe setosa* increased [46]. Thus, tomato leaf curling may be a potentially useful trait to improve tomato resistance to drought under field conditions.

Before rice leaves curl, first the stomata of the epidermal cells will close upon initiation of water deficit [43]. Plants adapted to dry conditions close their stomates after most mesophytes have already closed them once water deficit has developed, whereby photosynthesis does not stop immediately in these plants [46]. SlARF4-as plants have been used to study stomatal conductance, and they have shown lower stomatal conductance than that of their WT counterparts. However, stomatal conductance of the SlARF4-as phenotype did not change significantly under salt or Polyethylene glycol (PEG)-simulated water stress, compared with the control. Nonetheless, the WT showed a normal response to osmotic stress, with a significant decrease in stomatal conductance [47]. In our study, the epidermis of the upper leaves on *arf4* plants showed smaller stomata than that of the WT leaves, and they did not immediately close upon initiation of the water deficit treatment; furthermore, the guard cells still retained their normal plumpness.

Vascular tissues provide mechanical support and are the means for water, nutrient, and hormone short- and long-distance transport in plants [48]. Directed cell division is an important factor in the development of vascular tissue; however, controlling the direction of division in space and time is a complex issue [48]. Auxin plays an important role in plant vascular activities; furthermore, auxin has a coordinated relationship with many hormones [49]. Auxin signal transduction and transport can affect the differentiation of vascular tissue, xylem formation, and improved water transport [25,50,51]. In our experiments, among the 628 genes specifically expressed in *arf4* mutants under water deficit stress, the phenylpropanoid biosynthesis pathway was one of the most enriched pathways. The phenylpropanoid biosynthesis pathway promotes the synthesis of lignin and responds to various biotic and abiotic stress conditions [52]. Lignin content is also closely related to the development of the far and near axis of mesophyll cells and vascular bundles [53]. Part of the auxin signal transduction pathway of *arf4* mutants was interrupted, and the stem thickness of *arf4* tomato plants was increased. Further observation through paraffin sections showed that *arf4* mutants had a more developed xylem than that in the WT plants. Therefore, stem thickening in *arf4* mutants may be related to the active lignin formation, which implies that the stem has a higher ability to transport water. As drought-resistant varieties have thick stems [42], the increased resistance of *arf4* mutants to water deficit may be related to the growth activity involved in the thickening of the stem.

Plant water status is another important indicator for evaluating plant resistance to water deficit [6]. In the experiments reported here, the rate of water loss in *arf4* leaves was lower than that recorded for the WT leaves. On the one hand, it benefited from leaf curling, which reduced transpiration, whereas at the same time, the stomata did not close completely, thereby maintaining transpiration activity; on the other hand, compared with the WT plants, water loss was slow, which was also reflected by the lower free water content. The rate of water loss in *arf4* large leaves with petioles was higher than that of the WT leaves, which was closely related to the more developed xylem in *arf4*, whereby the latter was able to hold more water and, thus, maintain an enduring hydrated status [54].

Water deficit causes a large amount of ROS to be produced in and out of plant cells. ROS can act as signal molecules to promote stomatal closure; furthermore, they can cause damage to the cell membrane structure and protein denaturation and trigger programmed cell death [55]. Sustained ROS accumulation will have a negative effect on normal cellular physiological and biochemical activities. For example, after water stress, sustained ROS presence causes premature leaf aging, whereby photosynthetic physiology cannot be restored and, consequently, the plant dies [56]. In this study, the maximum fluorescence intensity of tomato plants after water stress revealed that WT tomato plants were irreversibly damaged. The actual photosynthetic efficiency of WT plants decreased significantly under water stress, whereas the decrease in *arf4* mutants was not significant. Additionally, *arf4* mutants resumed active growth upon re-watering for 24 h, whereas WT plants continued to wither. Maximum fluorescence intensity in WT leaves seemed irreversible, reflecting that WT plants were still under stress despite re-watering, implying that the rehydration capacities of *arf4* and WT plants were significantly different. We believe that *arf4* improves the rehydration ability of plants after stress relief and confers stronger resistance to water deficit. Furthermore, tomato plants can adapt to water stress by reducing oxidative damage [57]. Here, the superoxide anion production rate in *arf4* plants was significantly higher than that in the WT plants under water stress. However, cell membrane oxidative damage was lower in *arf4* plants because of its higher antioxidant capacity, which explains why *arf4* plants showed greater resistance to water deficit [58,59,60].

**Figure 6:**
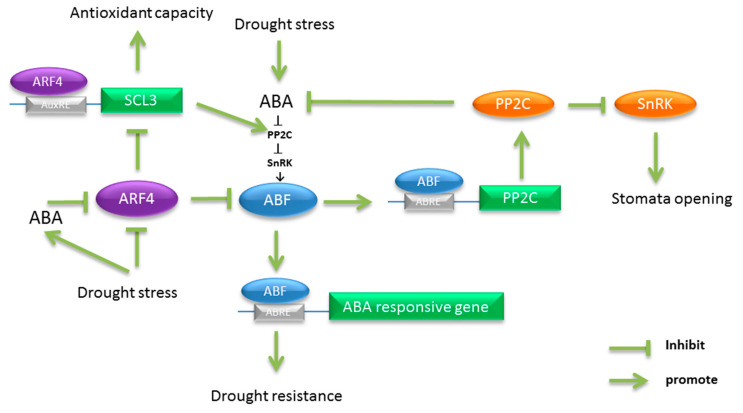
Hypothetical model of *SlARF4* participation in ABA signal transduction pathway to regulate resistance to water deficit in tomato plants.

The transcriptome and molecular data acquired in this study showed that the ABA signaling pathway played an important role in *arf4* plant resistance to water deficit. Recent studies have shown that ABA can induce the upregulation of *ABF* expression, resulting in upregulating the expression of *PP2Cs* to inhibit downstream SnRK2 phosphorylation, which negatively regulates the ABA signaling pathway. At the same time, *ABF* can be further combined with related genes induced by ABA [61]. In Arabidopsis, overexpression of *ABF* can directly induce the expression of ABA downstream-related genes without ABA processing [62]. This implies that ABF can independently regulate the expression of ABA-induced genes in plants. Overexpression of *ABF* can enhance plant resistance to water stress, whereas the *ABF*-deletion mutant reduces it [63,64,65]. In our experiments, both water deficit and ABA treatments reduced the expression of the *ARF4* gene in tomato. The expression levels of *ABI5* (*ABF*) in *arf4* plants under water stress increased significantly. In the promoters of *ABI5* there is AuxRE element indicating that SlARF4 could directly regulate *ABI5* expression. Meanwhile, anther transcription factor SCL3, also named *GRAS4*, was reported to improve water deficit resistance by regulating ABA signaling via modulating SnRK expression in tomato directly [66]. In our study, the expression of *SCL3* was up-regulated in the *arf4*. Furthermore, two AuxRE elements were found in its promoter indicating that SlARF4 improved water deficit resistance by regulating *SCL3*. In this study, *SlARF4* was found to accumulate in leaf stomata by GUS staining, and the stomata on the leaf epidermis of *arf4* mutant plants could not close normally under water stress, implying an important role of SlARF4 in stomata movement under water stress. Based on these findings, a possible regulation mechanism is proposed in Figure 6. Plant homeostasis plays an important role in plant resistance to stress. Hormonal responses in plants aim to maintain a dynamic balance under stress. Specifically, under water stress, ABA is transported to the guard cells. ABA promotes the accumulation of ABF by regulating the activity of SnRK2, whereas the auxin responsive protein ARF4 can inhibit the expression of *ABF* under water stress. This reversed function keeps ABF concentration in a dynamic equilibrium to prevent an excessive expression of *ABF*, which would eventually close stomata. However, in *arf4* plants, the deletion of SlARF4 significantly upregulates the expression of *ABF*, promotes the massive expression of the *PP2C* family, dephosphorylates SnRK, and cannot further regulate the expression of stoma-related genes, whereby stomates are rendered unable to close.

## 4. Materials and Methods

### 4.1. Plant Material and Water Deficit Treatment

The *SlARF4* knockout mutants by CRISPR/Cas 9 were provided by the lab of GBF, Université de Toulouse, INRA. Two single-guide (sg) RNAs (AATGGAGGTCACACCAGAG and GGAACTGAA AAGCCACCAT) in the coding sequence of Solyc11g069190 were designed and cloned into the vector pAGM4723. The positive construct was transformed into *Agrobacterium tumefasciens* which was used for tomato genetic transformation. The plant #5 bearing the desired mutation of SlARF4 was used for the water deficit study. More information about the SlARF4 CRISPR/Cas 9 lines generation could be found in the paper of Bouzroud et al. [47]. Tomato SlARF4 knockout (*arf4*) and pARF4::GUS plants were grown in a controlled climate room (25 ± 5 °C) under a 16 h/8 h (light/dark) photoperiod at the South China Agricultural University. Tomato plants were either kept under conditions of unlimited water supply (control) or subjected to a water deficit for 10–15 days; each treatment included three biological replicates. The leaves at the same position were collected from each replicate of both control and water stress treatments. Leaf samples were immediately frozen in liquid nitrogen and stored at −80 °C until analysis. MS medium containing 100, 200, or 300 μM mannitol was prepared to simulate water deficit. Tomato seedlings of WT and transgenic plants were incubated aseptically for 2 weeks to measure the root and hypocotyl length.

### 4.2. RNA-seq Analysis

All samples (three biological replicates) were sent to Guangzhou Gene Denovo Biological Technology Co., Ltd. (Guangzhou, China) for RNA isolation and RNA-Seq library preparation and sequencing. The cDNA libraries were sequenced using the Illumina HiSeqTM 2500. Sequence read mapping and assembly were performed using the procedure described by Song [67]. DEGs were determined using an FDR < 0.05 and an absolute value of |log2 (fold change)| > 1 as the threshold. The KEGG database was used to identify putative biological functions and pathways according to Mao et al. [68]. Transcriptome data analysis and mapping were carried out using OmicShare Tools (www.omicshare.com/tools) (accessed on 26 January 2021), a free online platform developed by Guangzhou GENE DENOVO Biotech.

### 4.3. Paraffin Transverse Section of Stem Tissues

Plant material was immersed in Formalin-Aceto-Alcohol (FAA) fixing solution. Vacuum was applied for 15–20 min and then the tissues were stored in 70% alcohol after fixation for 1–2 days. A 30–95% gradient alcohol dehydration treatment was carried out for one hour at each stepwise increase of alcohol concentration. Tissue sections were subsequently immersed in ethanol:xylene (1:1) transparent treatment and xylene:paraffin (1:1) mixture for 13–20 h and then soaked in pure paraffin three times for 1 h each time. Subsequently, these tissues were embedded and sectioned and dewaxed twice with pure xylene for 10 min each time, which was followed by rehydration treatment with each alcohol concentration. Finally, toluidine blue or eosin aqueous solution dyeing were applied for 30 min to 1 h, and tissues were then thoroughly rinsed and observed after dehydration treatment with 70–100% ethanol.

### 4.4. GUS Staining

The GUS staining solution included 50 μM sodium phosphate buffer solution (pH 7.2), 2 mM K_4_Fe_6_, 0.2% Triton X-100, and 2 mM X-gluc. Leaves or seedlings were immersed in a recessed container containing GUS dye solution and vacuum-pumped at intervals of 5–5-10 min and then dyed in the dark at 37 °C for 24 to 48 h. Finally, an ethanol gradient was used to decolor until only GUS color was left.

### 4.5. Determination of Biochemical and Physiological Traits

Mature leaves were detached to determine the rate of water loss using the following formula: [water loss rate (%) = (weight at the 0 h weight at the nth h)/weight at the 0 h × 100%]. Leaf curl was calculated using the following formula [Leaf curl (%) = (maximum leaf width − natural leaf width)/maximum leaf width × 100%]. Stem thickness of the third internode from the ground on 2-month-old tomato plant was measured using a Vernier caliper. Observation of stomata was carried out on mature leaves between the 4th and 7th nodes from the shoot base in both control and treated plants. Image Pro was used for measuring upper and middle stomata-related data. Measurements were performed between 09:00 and 11:00 h using a microscope BX53 (Olympus, Japan). Superoxide anion production rate, antioxidant enzyme activities (SOD, POD, and CAT), and malondialdehyde (MDA), ascorbic acid, and glutathione contents were measured following the methods described by Loukehaich et al. [69].

The ImageJ software (Image-Pro Plus 6.0) was used to measure hypocotyl and root length. Three technical repeats were performed for all quantifications.

### 4.6. Analysis of Chlorophyll Fluorescence

Maximal quantum yield of PSII (Fv/Fm) and actual photochemical efficiency of PSII (YII) have been widely used to reflect drought resistance in vegetables [70,71]. In this study, the Fv/Fm and YII were measured by using an imaging-PAM fluorometer (Walz, Effeltrich, Germany). The two-month-old tomato plants were darkened for 30 min prior to measurement. The third fully expanded leaves from the top were picked, smoothed and placed on the table of the chlorophyll fluorescence imaging system. First, initial fluorescence (Fo) was measured during the weak measuring pulses and then maximal fluorescence (Fm) was measured by a 0.8 s pulse light, and images for chlorophyll fluorescence were taken at the same time. Next, the actinic light was used to stimulate normal photosynthesis for several minutes. During illumination, steady-state fluorescence (Fs) and maximal fluorescence in this light (Fm’) were obtained. Fv/Fm was calculated using the equation: Fv/Fm = (Fm − Fo)/Fm. YII was calculated using the equation: Y(II) = (Fm’ − Fs)/Fm’. Three leaves for one biological repetition and three biological repetitions were measured in this study.

### 4.7. Statistical Analysis

The data presented were the mean of three replications with corresponding standard errors (mean ± SE). Data were analysed using IBM SPSS statistics 23 software (SPSS Inc. Chicago, IL, USA). The one-way analysis of variance (ANOVA) was carried out on the SPSS software. The differences between the means were determined by Tukey’s least significant difference (LSD) test at *p* < 0.05. The value with different letters was considered a significant difference at *p* < 0.05.

### 4.8. RNA Isolation and qRT-PCR Analysis

To validate the RNA-seq results, water-stressed tomato leaf samples from each sampling time point were subjected to qRT-PCR analysis. Total RNA was provided by Gene Denovo Biological Technology Co., Ltd. (Guangzhou, China). The cDNA was reverse-transcribed using the PrimeScript RT Reagent Kit with gDNA Eraser (Takara, China), following the protocol of the manufacturer. Gene-specific qRT-PCR primers were designed using Primer-BLASÉ in National Center for Biotechnology Information (NCBI) (https://www.ncbi.nlm.nih.gov/) (accessed on 10 January 2019) for 13 selected genes. qRT-PCR was performed using a LightCycler-480 RT-PCR system (Roche, Basel, Switzerland). Each reaction mixture contained 5 µL 2 × TB Green Master Mix Reagent (Takara, China), 1 µL cDNA sample, and 100 nM gene-specific primer in a final volume of 10 µL. PCR conditions were as follows: 95 °C for 30 s, followed by 40 cycles of heating at 95 °C for 5 s and annealing at 60 °C for 30 s. A template-free control for each primer pair was set for each cycle. All PCR reactions were normalized using the Ct value corresponding to the tomato UBI gene. Measurements of three biological and three technical replicates were used.

## 5. Conclusions

SlARF4 is involved in the growth and development of tomato plants. Under water deficit, SlARF4 participates in ABA signaling pathways by regulating *ABI5/ABF* and *SCL3* expression, and by influencing stomatal morphology and vascular bundle development to improve plant resistance to water deficit.

## Data Availability

Data available in a publicly accessible repository.

## Supplementary Materials

The following are available online at https://www.mdpi.com/1422-0067/22/7/3347/s1, Figure S1. Morphology of water stress-resistant tomato seedlings under mannitol treatment. (A) WT and *arf4* mutant tomato seed germination rate; (B) Hypocotyl length in WT and *arf4* mutant tomato seedlings cultured during 2 weeks in 100 and 300 μM mannitol-supplemented MS medium; (C) Root length of WT and *arf4* mutant tomato seedlings cultured during 2 weeks at 100 and 300 μM mannitol-supplemented MS medium. (D) Upper and lower rows show 2-week-old WT and arf4 mutant tomato seedlings, respectively. The scale represents 1 cm. Significance level set at *p* < 0.05, *n* = 5. Figure S2. Correlation analysis of the expression of three biological repeated samples in tomato under different treatments. The WT and *arf4* (ARF4) mutant grown under water stress are represented by WT-D and arf4-D (ARF4-D), respectively. Figure S3. Gene expression associated with plant hormone signal transduction pathways in *arf4* mutants grown under water stress. The red box represents differently expressed genes. Figure S4. Gene expression of *arf4* mutants in Phenylpropanoid biosynthesis pathway under water stress. The red box represents differently expressed genes. Figure S5. Two set of specific differentially expressed genes involved in ABA and salicylic acid (SA) signaling pathways. The red box represents differently genes. Figure S6. Auxin response element analysis of 3000 bp upstream promoters’ sequences of *ABi5*, *SCL3*, *EXO* and *solyc07g055460.3.* Figure S6. Comparison between fluorescent qRT-PCR and transcriptome sequencing results for 13 selected genes. ARF4 represents the *arf4* mutant. Figure S7. Pearson’s correlation between RNA-seq data and qRT-PCR data. Table S1. Effect of water stress on stomata morphology of tomato leaf epidermis. Table S2. Summary statistics of RNA-seq data of the 12 libraries mapped to the tomato reference genome (*Solanum lycopersicum ITAG2.3*). Table S3. KEGG pathways analysis of 628 DEGs. Table S4. KEGG pathways analysis of 86 DEGs. Table S5. KEGG pathways analysis of 32 DEGs.
