# Supplementary material for: Knockout of Auxin Response Factor SlARF4 Improves Tomato Resistance to Water Deficit
Source: Int J Mol Sci. 2021 Mar 25;22(7):3347. doi: 10.3390/ijms22073347 (PMC8037468; doi:10.3390/ijms22073347)
Supplement: Supplementary file 1 [file ijms-22-03347-s001.zip › supplementary files/Table S1.docx]

**Table S1.** Effect of water stress on stomata morphology of tomato leaf epidermis.

Different letters (a, b, c, d) present significant difference at level set p < 0.05.

| Treatment | | Stomata length(μm) | Stomata width(μm) | Ratio of the length to width of stomata | Stomata aperture | |
| --- | --- | --- | --- | --- | --- | --- |
|  |  |  |  |  | Length(μm) | Width(μm) |
| Top | WT | 118.18±4.279 a | 87.83±2.07 a | 1.35±0.057 b | 66.38±1.55 a | 23.36±3.43 a |
|  | *arf4* | 114.85±0.589 a | 88.07±3.14 a | 1.31±0.063 b | 55.82±1.41 b | 18.69±0.86 ab |
|  | WT-Drought | 118.18±2.08 a | 72.99±3.14 b | 1.63±0.055 a | 67.88±1.05 a | 14.90±1.34 b |
|  | *arf4*-Drought | 102.92±2.11 b | 70.78±2.22 b | 1.46±0.057 b | 50.47±1.75 c | 13.19±1.84 b |
|  |  |  |  |  |  |  |
| Middle | WT | 122.22±3.50 b | 75.02±2.14 b | 1.63±0.049 ab | 74.18±4.03 b | 16.21±1.43 a |
|  | *arf4* | 123.89±0.88 b | 84.71±4.19 a | 1.47±0.058 b | 67.57±0.30 c | 17.84±1.21 a |
|  | WT-Drought | 138.12±1.59 a | 83.90±1.24 a | 1.65±0.034 a | 90.75±0.56 a | 15.41±0.50 a |
|  | *arf4*-Drought | 119.96±2.68 b | 80.08±1.98 ab | 1.50±0.059 ab | 65.69±0.80 c | 13.86±1.99 a |
