# Supplementary material for: Knockout of Auxin Response Factor SlARF4 Improves Tomato Resistance to Water Deficit
Source: Int J Mol Sci. 2021 Mar 25;22(7):3347. doi: 10.3390/ijms22073347 (PMC8037468; doi:10.3390/ijms22073347)
Supplement: Supplementary file 1 [file ijms-22-03347-s001.zip › supplementary files/Table S2.docx]

**Table S2.** Summary statistics of RNA-seq data of the 12 libraries mapped to the tomato reference genome (*Solanum lycopersicum ITAG2.3*).

| Samples | WT | | | *arf4* | | | WT-D | | | *arf4*-D | | |
| --- | --- | --- | --- | --- | --- | --- | --- | --- | --- | --- | --- | --- |
| Library | WT-1 | WT-2 | WT-3 | *arf4*-1 | *arf4*-2 | *arf4*-3 | WT-D-1 | WT-D-2 | WT-D-3 | *arf4*-D-1 | *arf4*-D-2 | *arf4*-D-3 |
| Clean reads | 61,740,434 | 50,748,450 | 59,765,904 | 63,139,362 | 60,807,576 | 65,209,156 | 69,945,882 | 55,400,718 | 62,073,974 | 48,483,202 | 51,265,354 | 47,929,570 |
| High quality clean reads (%) | 60,633,380 (98.21%) | 49,894,320 (98.32%) | 58,743,370 (98.29%) | 62,290,954 (98.66%) | 60,024,184 (98.71%) | 64,359,344 (98.7%) | 68,668,208 (98.17%) | 54,387,410 (98.17%) | 61,022,670 (98.31%) | 47,876,728 (98.75%) | 50,526,036 (98.56%) | 47,334,244 (98.76%) |
| Removed rRNA reads (%) | 59,995,818 ( 98.95% ) | 48,853,860 ( 97.91% ) | 57,564,412 ( 97.99% ) | 61,472,050 ( 98.69% ) | 59,597,824 ( 99.29% ) | 63,778,226 ( 99.10% ) | 67,963,734 ( 98.97% ) | 53,106,068 ( 97.64% ) | 60,055,974 ( 98.42% ) | 46,850,926 ( 97.86% ) | 49,233,216 ( 97.44% ) | 46,702,726 ( 98.67% ) |
| Mapped reads(%) | 55,373,849(92.30%) | 45,116,457  (92.35%) | 53,284,719(92.57%) | 56,974,577(92.68%) | 55,490,743(93.11%) | 59,406,422(93.15%) | 61,538,961(90.55%) | 48,609,263(91.53%) | 54,595,075(90.91%) | 43,598,773(93.06%) | 45,087,063  (91.58%) | 43,071,054  (92.22%) |
| Unique mapped reads (%) | 54,918,117 (91.54%) | 44,739,859 (91.58%) | 52,853,441 (91.82%) | 56,483,935 (91.89%) | 55,029,087 (92.33%) | 58,914,434 (92.37%) | 61,139,357 (89.96%) | 48,322,651 (90.99%) | 54,221,395 (90.28%) | 43,280,777 (92.38%) | 44,758,237 (90.91%) | 42,765,290 (91.57%) |
| Multiple mapped reads | 455,732 (0.76%) | 376,598 (0.77%) | 431,278 (0.75%) | 490,642 (0.80%) | 461,656 (0.77%) | 491,988 (0.77%) | 399,604 (0.59%) | 286,612 (0.54%) | 373,680 (0.62%) | 317,996 (0.68%) | 328,826 (0.67%) | 305,764 (0.65%) |
| All genes | 22,868 | 22,721 | 22,820 | 22,702 | 22,709 | 22,894 | 22,779 | 22,486 | 22,588 | 22,619 | 22,567 | 2,2677 |
| Known genes (%) | 23,599 (65.98%) | | | 23,507 (65.72%) | | | 23,405 (65.44%) | | | 23,347 (65.27%) | | |
| Novel transcripts | 607 | | | 601 | | | 616 | | | 599 | | |
