# Supplementary material for: Knockout of Auxin Response Factor SlARF4 Improves Tomato Resistance to Water Deficit
Source: Int J Mol Sci. 2021 Mar 25;22(7):3347. doi: 10.3390/ijms22073347 (PMC8037468; doi:10.3390/ijms22073347)
Supplement: Supplementary file 1 [file ijms-22-03347-s001.zip › supplementary files/supplementary figures.docx]

Figure S1. Morphology of water stress-resistant tomato seedlings under mannitol treatment. (A) WT and *arf4* mutant tomato seed germination rate; (B) Hypocotyl length in WT and *arf4* mutant tomato seedlings cultured during 2 weeks in 100 and 300 μM mannitol-supplemented MS medium; (C) Root length of WT and *arf4* mutant tomato seedlings cultured during 2 weeks at 100 and 300 μM mannitol-supplemented MS medium. (D) Upper and lower rows show 2-week-old WT and arf4 mutant tomato seedlings, respectively. The scale represents 1 cm. Significance level set at *p* < 0.05, *n* = 5.

Figure S2. Correlation analysis of the expression of three biological repeated samples in tomato under different treatments. The WT and *arf4* (ARF4) mutant grown under water stress are represented by WT-D and arf4-D (ARF4-D), respectively.

Figure S3. Gene expression associated with plant hormone signal transduction pathways in *arf4* mutants grown under water stress. The red box represents differently expressed genes.

Figure S4. Gene expression of *arf4* mutants in Phenylpropanoid biosynthesis pathway under water stress. The red box represents differently expressed genes.

Figure S5. Two set of specific differentially expressed genes involved in ABA and salicylic acid (SA) signaling pathways. The red box represents differently genes.

Figure S6. Auxin response element analysis of 3000 bp upstream promoters’ sequences of *ABi5*, *SCL3*, *EXO* and *solyc07g055460.3.*

Figure S7. Comparison between fluorescent qRT-PCR and transcriptome sequencing results for 13 selected genes. ARF4 represents the *arf4* mutant.

Figure S8. Pearson’s correlation between RNA-seq data and qRT-PCR data.
